# Equine-assisted services for individuals with substance use disorders: a scoping review

**DOI:** 10.1186/s13011-022-00506-x

**Published:** 2022-12-14

**Authors:** Liam Diaz, Mirinda Ann Gormley, Ashley Coleman, Abby Sepanski, Heather Corley, Angelica Perez, Alain H. Litwin

**Affiliations:** 1 Addiction Medicine Center, Prisma Health, 876 W Faris Rd, Greenville, SC 29605 USA; 2 Department of Emergency Medicine, Prisma Health, 701 Grove Rd, Greenville, SC 29605 USA; 3 Department of Public Health Sciences, Clemson University College of Behavioral, Social, and Health Science, 503 Edwards Hall, Clemson, SC 29631 USA; 4 Department of Emergency Medicine, University of South Carolina School of Medicine- Greenville, 701 Grove Rd, Greenville, SC 29605 USA; 5 Lumin Center, 306A Mills Ave, Greenville, SC 29605 USA; 6 Clemson University School of Health Research, 605 Grove Rd., Suite 301, Greenville, SC 29605 USA; 7 Department of Medicine, University of South Carolina School of Medicine- Greenville, 701 Grove Rd, Greenville, SC 29605 USA; 8 Department of Internal Medicine, Prisma Health, 876 W Faris Rd, Greenville, SC 29605 USA

**Keywords:** Equine-assisted services, Horse-assisted therapy, Equine-assisted psychotherapy, Substance use disorder, Substance use disorder treatment

## Abstract

The implementation of equine-assisted services (EAS) during treatment for psychological disorders has been frequently documented; however, little is known about the effect of EAS on outcomes for populations with substance use disorder (SUD). The purpose of this scoping review was to synthesize existing literature reporting the effects of EAS when incorporated into SUD treatment. This review followed guidelines in the Preferred Reporting Items for Systematic Reviews and Meta-Analyses (PRISMA) checklist for scoping reviews. A search of four databases (MEDLINE/PubMed, PsycINFO, the Cumulative Index to Nursing and Allied Health Literature (CINAHL), and Academic OneFile) identified studies reporting quantitative and/or qualitative data for an EAS intervention that was implemented among a population receiving treatment for and/or diagnosed with SUD. Of 188 titles, 71 underwent full-text assessment and six met inclusion criteria. Three additional articles were identified through other sources. Quantitative data suggested a potential positive effect of EAS on SUD treatment retention and completion as well as participants’ mental health; however, studies were limited by small sample sizes. Qualitative data indicated that in addition to participants developing bonds with the horses and perceiving EAS as welcomed breaks from traditional treatment, they experienced increased self-efficacy, positive emotional affect, and SUD treatment motivation. Due to methodological limitations and an overall dearth of available studies, the effectiveness of EAS as adjuncts to SUD treatment is inconclusive. Additional research is necessary before supporting broad implementation of EAS as adjuncts to SUD treatment.

**Supplementary Information:**

The online version contains supplementary material available at 10.1186/s13011-022-00506-x.

## Introduction

In 2020, 40.3 million Americans recorded a past year substance use disorder (SUD), yet only approximately one-tenth of this number received any specialty treatment [1]. Among those who do engage in SUD care, retention is uncertain and dropout rates across in-person psychosocial treatments average 30.4% [2]. Given the documented positive association between time in treatment and improved outcomes for individuals with SUD [3–5], there is a need to identify interventions that can be used to increase treatment motivation, retention, and remission.

One potential avenue for such interventions is equine-assisted services (EAS). EAS is a unifying term that captures all services where professionals incorporate horses (or equines) to benefit people [6]. Of particular interest are psychotherapies incorporating equines, such as equine-assisted psychotherapy (EAP) and equine-facilitated psychotherapy (EFP). With these novel and complementary approaches to psychotherapy, the horse serves as a supporter of psychotherapeutic interventions as well as a mediator between psychotherapist and client [7]. In this therapeutic triad (client-horse-therapist), by prompting specific behaviors and serving as a metaphor for understanding internal experiences, the horse facilitates personal exploration and provides a context for interpreting client difficulties [8]. Horses may be suited for this therapeutic role due in part to a keen awareness of and sensitivity to nonverbal communication [8, 9]. Horses’ automatic responses to client behaviors provide valuable information that can inspire client insight and self-awareness [8, 9]. Additionally, horses are claimed to be well-positioned due to their powerful physical attributes and the resulting emotions they stir in people [9, 10].

The implementation of equines during treatment for psychological disorders has been frequently documented. Following EAS, participants have reported significant reductions in symptoms of psychological distress [11], hyperactive behaviors [12], violent behaviors [13], post-traumatic stress disorder (PTSD) [14, 15], and anxiety [15, 16].

In line with this developing body of research, there have been media reports of incorporating equines into SUD treatment [17–19]. However, while some individuals have indicated a beneficial effect of EAS on substance misuse [20], few studies have provided experimental/non-anecdotal support for their effectiveness on the recovery process. One study evaluating the efficacy of an EAS intervention for treating anxiety and PTSD found a significant reduction in alcohol use among participants [15].

While previous research has indicated a potentially beneficial effect of EAS on behavioral health outcomes, there remain significant concerns regarding methodological limitations of corresponding studies and the possibility of marketing expensive treatments of questionable efficacy and challenging scalability to vulnerable populations [8]. Therefore, the purpose of this scoping review is to discuss the current state of the literature, identify gaps in knowledge, and propose future directions for research necessary to accurately evaluate the effectiveness of EAS on recovery-related outcomes for individuals undergoing SUD treatment. A scoping review design was selected due to an anticipated lack of published literature on the use of EAS in SUD recovery and a desire to comprehensively identify all existing studies.

## Methods

### Information sources

This scoping review followed guidelines in the PRISMA checklist for scoping reviews [21]. The literature search included four databases: MEDLINE/PubMed, PsycINFO, the Cumulative Index to Nursing and Allied Health Literature (CINAHL), and Academic OneFile. Academic OneFile was queried to increase the opportunity to identify eligible studies within gray or lay literature. Databases were searched from database inception to July 27^th^, 2021. At this time, scoping review protocols cannot be registered with PROSPERO and the protocol was not published elsewhere.

### Search criteria

The search query included the medical subject heading “substance-related disorders,” SUD-related keywords, and EAS-related keywords. A full description of the search terms can be found in Supplemental Table 1. In addition to the database search, one author (MAG) conducted a thorough search of past systematic reviews to identify any potentially relevant articles not produced in the database search.

### Eligibility criteria

Articles were included in this review if they reported quantitative and/or qualitative data for an EAS intervention that was implemented among a population receiving treatment for and/or diagnosed with a SUD. Studies were excluded if the EAS intervention was implemented in a population not comprised of those receiving treatment for and/or diagnosed with a SUD. Studies exclusively focusing on hippotherapy were excluded due to hippotherapy’s designation as a therapy tool for physical, occupational, and/or speech therapy and its typical use in populations with neuromotor and/or cognitive/communication deficits [22].

### Selection of evidence

Sequential screening of article titles, abstracts, and full-text articles was performed independently by two reviewers (LD, MAG). Any disagreements were resolved through consensus. Data extraction was performed independently by two authors (LD, MAG) using originally developed Microsoft Excel spreadsheets. Information extracted on the study characteristics included: first author, publication year, study objective, study design, study setting, years of recruitment, population, sample size, description of study groups, study outcomes or qualitative themes, subthemes, and brief results. Information extracted on EAS interventions included: name of EAS intervention, definition of EAS intervention, EAS staff qualifications, EAS activities, and descriptions of EAS activities. Disagreement during the data extraction process was resolved through consensus. Following data extraction, quantitative and qualitative data were jointly reviewed and qualitatively synthesized/summarized by two authors (LD, MAG).

## Results

**Fig. 1:**
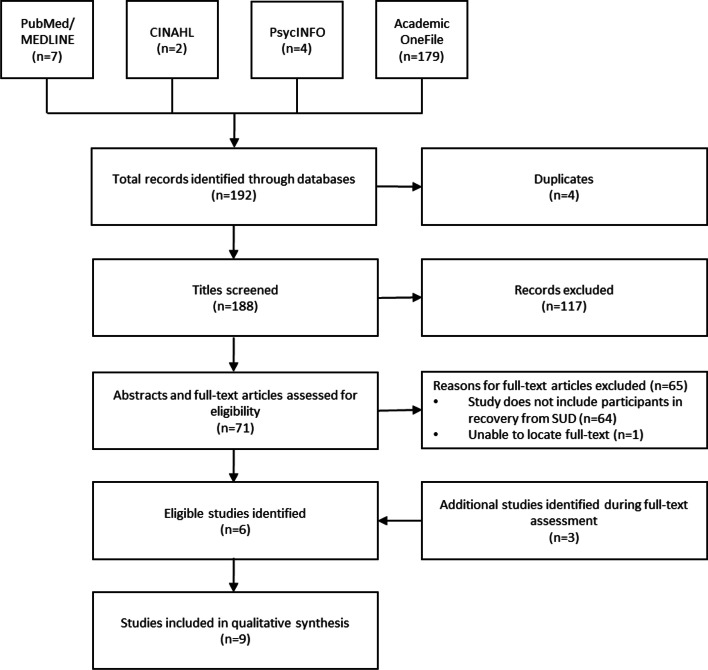
Database search and selection of eligible articles

The search yielded a total of 192 articles (Fig. 1). Of the 188 titles that remained after the removal of duplicates (*n* = 4), 71 were selected for full-text assessment. The majority of titles, largely comprised of magazine and news media articles, did not include abstracts. Therefore, all articles selected during title assessment went through full-text assessment. Of those 71 titles, 65 were removed, either due to an inability to locate the full-text article (*n* = 1) or because the article, or study referenced within the article, did not include participants diagnosed with and/or in treatment for a SUD (*n* = 64). In addition to the six studies remaining after full-text assessment, two studies [23, 24] were discovered searching the references of these papers and one study [25] was found via a more recent article search on November 11^th^, 2021, yielding nine total studies for qualitative synthesis.

### Study settings and populations

Table 1 summarizes the study and intervention-related characteristics of corresponding EAS. Six studies were qualitative [23, 24, 26–29]. The three quantitative studies included a randomized control trial [30], prospective cohort analysis [31], and quasi-experimental study [25]. All but two studies were peer-reviewed publications, one study was an unpublished master’s thesis [23] and one was a doctoral dissertation [29]. Five of the studies took place in Norway [23, 27, 28, 30, 31], two in Canada [24, 26], one in the United States [25], and one did not specify location [29].

**Table 1:** Study and intervention characteristics of equine-assisted services incorporated into substance use disorder treatment

**Author, Year**	**Setting and Years of Recruitment**	**Study Design** **(*n*)**	**EAS Intervention**	**EAS Staff -** **Qualifications**	**Duration of Treatment**	**Horse Activities**	**Treatment Objectives**
**Adams et al., 2015 ** [26]	First Nations female youths attending the Cartier Equine-Assisted Learning program in Saskatchewan, Canada as part of their residential treatment for volatile substance misuse between June 2010 and June 2012.	Qualitative *n *= 26	Equine-Assisted Learning (EAL)	EAL Program Facilitator	20 weeks	Non-riding/ground activities.	Participant self-development; internalization and generalization of awareness developed during EAL sessions to other life situations; cognitive and behavioral change.
**Atherton et al., 2020 ** [25]	Adolescents with an active diagnosis of a SUD referred to the study by a local resident provider in a medium-sized rural city in the Southeastern US. Years of recruitment not provided.	Quasi-experimental *n* = 10	Equine-Facilitated Psychotherapy (EFP)	Addiction Counselor Equine Specialist Horse Trainer	6 weeks (1.5 h/week)	Horseman’s handshake; herd observation; grooming; groundwork (e.g., catching the horse, leading the horse around the arena, leading the horse through obstacle courses with and without a lead rope and halter, directing the horse to circle around the participant when attached to a long rope).	Each EFP session's theme and corresponding therapeutic and horsemanship goals were developed based on choice theory's connecting behaviors: listening, trusting, encouraging, respecting and accepting, and negotiating differences.
**Brenna et al., 2013 ** [23]	Individuals undergoing treatment at the Oslo University Hospital’s Department of Addiction treatment- Youth in Oslo, Norway. Years of recruitment not provided.	Qualitative *n* = 8	Horse-Assisted Therapy (HAT)	HAT Therapist- Experienced therapists that were also qualified riding instructors	12 sessions	Stable work (e.g., grooming and feeding); groundwork; mounted work.	Planned part of overall SUD treatment; agreed goals between therapist and participant (e.g., management of anxiety or aggression, need to set and maintain safe limits).
**Dell et al., 2011 ** [24]	First Nation and Inuit youth attending the Keystone Equine Center and the Lambton Equine Assisted Learning Centre in Ontario, Canda as part of their residential treatment for solvent abuse from October 2008 to April 2009.	Qualitative *n* = 15	Equine-Assisted Learning (EAL)	EAL Program Facilitator	12 weeks (1 h/week)	Non-riding activities.	Increasing self-esteem; modifying behavior; healing while having fun.
**Gatti et al., 2020 ** [30]	Adult inpatients with a primary diagnosis of mental and behavioral disorders due to psychoactive substance use (ICD-10) receiving treatment at the Department of Addiction Treatment (Youth) at Oslo University Hospital from January 2013 to January 2016.	Randomized Control Trial *n* = 37	Complementary Horse-Assisted Therapy (cHAT)	HAT Therapist- Qualified psychotherapists specializing in equine-facilitated psychotherapy	6 weeks (2 90-min sessions/week)	Herd behavior observation; stable work; groundwork; mounted work; vaulting; driving a carriage.	Planned part of overall SUD treatment; agreed goals between therapist and patient; Challenges related to SUDs (e.g., boundary setting, development of trust, control of emotional affect).
**Kern-Godal et al., 2015 ** [31]	Inpatients and day patients admitted to the Department of Addiction Treatment- Youth at Oslo University with a primary diagnosis of mental and behavioral disorders due to psychoactive substance use (ICD 10) between January 1, 2011 and June 30, 2012.	Prospective Cohort *n* = 108	Horse-Assisted Therapy (HAT)	HAT Therapist- Qualified therapists that were also Norwegian Level 1 Riding Instructors	6 weeks (2 90-min sessions/week)	Herd behavior observation; stable work; groundwork; mounted work; driving work.	Planned part of overall SUD treatment; agreed goals between therapist and patient (e.g., strengthen boundaries setting, reduce anxiety/depression/aggression).
**Kern-Godal, Brenna, Arnevik, et al., 2016 ** [27]	Individuals undergoing treatment at the Department of Addiction Treatment (Youth), Oslo University Hospital in Oslo, Norway between November 2012 and end of January 2013.	Qualitative *n* = 8	Horse-Assisted Therapy (HAT)	HAT Therapist- Experienced therapists that were also qualified riding instructors	12 sessions	*Not Provided*	Planned part of overall SUD treatment; agreed goals between participant and therapist.
**Kern-Godal, Brenna, Kogstad, et al., 2016 ** [28]	Individuals at a SUD treatment facility in Oslo, Norway who participated in at least one hour of HAT between 2012 and early 2013.	Qualitative *n* = 8	Horse-Assisted Therapy (HAT)	HAT Therapist- Experienced psychotherapists that were also riding instructors and skilled horse handlers	*Not Provided*	Grooming; feeding; riding; mucking out; moving hay; tack cleaning.	*Not Provided*
**Stiltner et al., 2013 ** [29]	Adolescent boys with dual diagnoses of substance abuse and mental disorders living at a residential treatment center that incorporates equine-assisted psychotherapy into its overall treatment program. Years of recruitment not provided.	Qualitative *n* = 8	Equine-Assisted Psychotherapy (EAP)	EAP Therapist- Therapists trained through the Equine Assisted Growth and Learning Association’s model Horse Professional Handler	*1 day/week*	Leading the horse; maneuvering the horse around obstacles; riding the horse.	*Not Provided*

*EAS* equine-assisted services, *SUD* substance use disorder

### Demographics and clinical characteristics

The majority of study participants were 26 years of age or younger with four studies including strictly adolescents [24–26, 29]. Less than half of the included studies provided demographic information on race or ethnicity [24–26, 29]. Five studies provided data on participant substance use [24, 26, 29–31]. Among these studies, a wide variety of substance use was characterized with no clear indication of a primary substance of misuse among populations receiving EAS. Four of the nine studies assessed for the presence of co-morbid mental health conditions among participants [28–31]. Additional demographic characteristics for each study are summarized in Supplemental Table 2.

### EAS-related results

EAS captured in this review included horse-assisted therapy (HAT) (*n* = 5) [23, 27, 28, 30, 31], equine-assisted learning (EAL) (*n* = 2) [24, 26], equine-assisted psychotherapy (EAP) (*n* = 1) [29], and equine-facilitated psychotherapy (EFP) (*n* = 1) [25].

EAS staff qualifications were documented in six of the nine studies (Table 1) [23, 27–31]. HAT therapists were consistently described as qualified/experienced psychotherapists with additional horsemanship-related competencies [23, 27, 28, 30, 31]. Stiltner [29] documented EAP therapists as being trained through the Equine Assisted Growth and Learning Association (EAGALA) model. For curriculum design, it appears that all five HAT studies employed the “Gaustad Model” of horse-assisted therapy at Oslo University Hospital (unpublished) [23, 27, 28, 30, 31]. For EAL, Adams et al. [26] detailed the Cartier Equine Learning Center as developing an “EAL formula” distinct from other programs and based on six components: relationship, curriculum, formula, horse, facilitation, and partnerships. Atherton et al. [25] described their EFP protocol as being developed according to a choice theory foundation with each session based on one of Glasser’s [32] connecting behaviors. Dell et al. [24] and Stiltner [29] did not document curriculum design.

Across EAS programs participants engaged in a variety of facilitator-led horse activities. In HAT, activities included various combinations of herd behavior observation, stable work, groundwork (such as grooming, leading, or setting limits), mounted work (riding in the arena, around the grounds, or in the woods), vaulting (gymnastics on horseback), and driving a carriage [23, 27, 28, 30, 31]. Each activity was described as having a different psychological significance and was incorporated at the discretion of the HAT therapist and according to patient treatment progression [30, 31]. For EFP, described activities largely included groundwork as well as the horseman’s handshake, herd observation, and grooming [25]. These activities were targeted toward specific therapeutic and horsemanship goals and each EFP session concluded with a half-hour of group processing facilitated by the intervention’s addiction counselor and equine specialist [25]. EAL activities were largely undescribed with the exception of programs being characterized as non-riding and focused on ground activities [24, 26]. Examples of EAP activities included leading the horse, maneuvering the horse around obstacles, and riding the horse [29].

### Quantitative results

#### Horse-assisted therapy

Two studies provided quantitative results for HAT (Table 2) [30, 31]. In both studies, treatment as usual (TAU) consisted of a person-centered program with both individual and group therapy that was based on a biopsychosocial model with emphasis on mentalization-based theory and practice. Using a naturalistic prospective cohort study design (*n* = 108), Kern-Godal et al. [31] reported that compared to TAU, HAT group participants were more likely to complete treatment (*p* < 0.001), remain in treatment for a longer period (*p* < 0.001), and remain in treatment for 90 days or more (aOR 3.9, *p* = 0.001). Using an intention-to-treat, randomized control trial (RCT) study design (*n* = 37), Gatti et al. [30] documented no significant association between participants receiving HAT versus TAU and dropout relative to treatment completion (OR: 0.60, *p* = 0.553) or transferring to another treatment facility relative to treatment completion (OR: 0.47, *p* = 0.335).

**Table 2:** Quantitative results for equine-assisted services incorporated into substance use disorder treatment

**Author, Year**	**Control Group**	**Outcome**	**Results**
**Atherton et al., 2020 ** [25]	Compared pretest and posttest measures after study participants completed 6-session equine-facilitated psychotherapy (EFP) group intervention.	**Adolescent Behavior Survey**	There was a statistically significant difference between participants' pretest and posttest results for all seven categories of the Adolescent Behavior Survey: “support of others,” “encouraging others,” “listen to others,” “accepting of others,” “trusting of others,” “respectful of others,” and “work cooperatively with others” (*p* < 0.001).
		**PHQ-9**	There was a statistically significant difference between participants' pretest (M = 17.00, SD = 2.16) and posttest (M = 10.20, SD = 1.55) PHQ-9 results (t(9) = 18.94, *p* < 0.001).
		**GAD-7**	There was a statistically significant difference between participants' pretest (M = 9.80, SD = 1.23) and posttest (M = 4.60, SD = 1.90) GAD-7 results (t(9) = 13.38, *p* < 0.001).
**Gatti et al., 2020 ** [30]	Compared to patients that received treatment as usual (TAU)^a^ only.	**Treatment Completion**	A non-statistically significant larger percentage of participants in the complementary horse-assisted therapy (cHAT) group completed their assigned program (44%) than the TAU-only group (32%).
		**Dropout**	Slightly fewer participants in the cHAT group dropped out of treatment (22%) than the TAU-only group (26%). There was no significant association between participants receiving intervention compared to TAU and dropout relative to completion (OR: 0.60, 95% CI 0.11–3.25, *p* = 0.553).
		**Transfer to Another Treatment**	Fewer participants in the cHAT group were transferred to another treatment (28%) than the TAU-only group (42%). There was no significant association between participants receiving intervention compared to TAU and transfer relative to completion (OR: 0.47, 95% CI 0.10–2.19, *p* = 0.335).
		**Time in Treatment**	There was no statistically significant difference in time in treatment between the cHAT group and the TAU-only group (mean 98.7 days, SD = 5.6 vs mean 107.4 days, SD = 23.65, *p* = 0.237).
		**Attendance at the HAT Program**	There was no statistically significant association between horse-assisted therapy (HAT) attendance (attending eight sessions or more) and treatment completion (OR: 3.000, 95% CI 0.211–42.65, *p* = 0.408).
**Kern-Godal et al., 2015 ** [31]	Compared to patients that received TAU^a^ only.	**Time in Treatment**	HAT participants remained in treatment for a significantly longer period of time than those who did not participate in HAT (mean 141 days, SD = 93.6 vs mean 70 days, SD = 73.8, *p* < 0.001). HAT participants were almost four times as likely to remain in treatment for 90 days or more (aOR 3.9 CI 1.7–8.8, *p *= 0.001).
		**Treatment Completion**	After excluding time in treatment and controlling for age, sex, education, number and severity of substances used, psychological distress and temporary exits, the aOR for HAT participants completing treatment was 8.4 (95% CI 2.7–26.4, *P* < 0.001).

^a^TAU consisted of a person-centered program with both individual and group therapy based on a biopsychosocial model with emphasis on mentalization-based theory and practice

#### Equine-facilitated psychotherapy

With a quasi-experimental, pretest–posttest design (*n* = 10), Atherton et al. [25] documented that after completing six EFP sessions, adolescents with an active SUD diagnosis experienced an increase in prosocial behaviors and a reduction in negative psychosocial symptoms, specifically depressive and anxious symptomology (*p* < 0.001) (Table 2).

### Qualitative results

Presented here is a synthesis of the common themes seen across qualitative studies (*n* = 6). Brenna [23] is not referenced in this synthesis due to overlap with qualitative results presented by Kern-Godal, Brenna, Arnevik, et al. [27] and Kern-Godal, Brenna, Kogstad, et al. [28], and the likelihood that these results are based on the same study population.

#### Developed bond/relationship with a horse

Four studies described the benefits of the bond or relationship participants formed with the horses [24, 26, 28, 29]. Many of the participants reported that bonding with the horses helped them develop trust [24, 26, 29]. These bonds likely formed because participants perceived the horses as non-judgmental [24, 28]. Participant reports included, “the horse accepts you, like, for who you are” [28]. Furthermore, in two studies interviewing First Nations and Inuit youth in Canada, researchers reported that working with the horses helped the youth develop a bond that facilitated spiritual exchange with their cultures [24, 26].

#### Increased self-efficacy and empowerment

All five studies reported that partaking in the EAS program empowered participants [24, 26–29]. Many participants reported increased self-efficacy through their “mastery” of working with the horses [28]. Dell et al. [24] reported that First Nations youth often shared their pride in how they learned to perform equine-specific techniques through communicative interactions with the horse. This increased confidence may also be due to the physicality associated with working with the horses, which gave participants a sense of purpose through meaningful engagement with a productive activity [27, 29].

#### Improved positive emotional affect

Three studies described perceived improvement in respondent mental health and emotional affect as a result of participation in the EAS programs [26, 28, 29]. Nearly all of the participants expressed happiness as well as a sense of calmness when engaging in EAS [26, 28, 29] and several participants reported that EAS had an effect on their mental health, particularly depression. In one study, a participant stated that upon leaving EAP, “I come out happy and not so depressed” [29].

#### Break from usual/traditional treatment

Another qualitative theme seen across studies is the feeling that EAS represented breaks from usual/traditional treatment for participants. Stiltner [29] reported that participants felt EAP made their residential facility seem less like an institution and credited the horses with making things feel “homey” [29]. In parallel, Kern-Godal, Brenna, Arnevik, et al. [27] documented participants describing HAT as a “pleasant variation” from usual treatment. Participants especially enjoyed the hands-on aspect of EAS versus traditional talk therapy [27, 29]. Dell et al. [24] detailed how EAL enhanced participants’ social wellbeing by providing participates an opportunity to participate in a new activity.

#### Treatment motivation

Across studies, participants additionally detailed the motivational impact of EAS on their treatment. Stiltner [29] documented how EAP participants looked forward to their sessions and wished that they could spend more time with the horses. Similarly, Kern-Godal, Brenna, Arnevik, et al. [27] described HAT as being a motivational factor for participants to come to treatment in the first place. Adams et al. [26] additionally documented that EAL participants experienced psychological healing as a result of increased participation and effort.

#### Negative/indifferent experiences

Four studies included in this review briefly reported on negative/indifferent participant experiences/attitudes regarding EAS as adjuncts to their SUD treatment. Kern-Godal, Brenna, Arnevik, et al. [27] detailed one participant as feeling that HAT had little to do with their motivation to succeed in treatment. Moreover, Stiltner [29] detailed two participants that did not look forward to their EAP sessions due to a fear of horses and displeasure with being outdoors. Lastly, Dell et al. [24] noted that several EAL participants complained that the equine facility was “cold” and “stinky like a farm.”

## Discussion

This scoping review is the first to synthesize literature discussing the implementation of EAS in populations diagnosed with or receiving treatment for SUDs. Results highlight potentially beneficial effects of EAS for individuals with SUDs; however, an overall dearth of studies and methodological limitations necessitate additional research before the implementation of EAS as adjuncts to SUD treatment can be supported.

Quantitative data summarized in the current study are insufficient to conclude a beneficial effect of EAS on the SUD recovery process. Using a more rigorous RCT-experimental design, Gatti et al. [30] was unable to reproduce the statistical significance of Kern-Godal et al. [31] and only observed nonsignificant differences in treatment completion (44% versus 32%) and time in treatment (98.7 days versus 107.4 days) for HAT participants compared to their TAU counterparts. Gatti et al. [30] suggests a reduction in sample size from *n* = 50, a value originally calculated based on findings from Kern-Godal et al. [31], to *n* = 37 as potentially responsible for the lack of statistical significance. Reasons for participant exclusion included: four were not eligible, two did not return informed consent documents, and seven withdrew consent based on randomization assignment. Continuing, among HAT participants, only 11% completed all 12 prescribed HAT sessions and among those who dropped out of SUD treatment 75% attended less than eight sessions. Gatti et al. [30] reasons that this may indicate a minimum number of sessions necessary to achieve meaningful effect when treating persons with SUDs. Despite the lack of statistical significance experienced by Gatti et al. [30], the suggestive positive effect of HAT on SUD treatment retention and completion seen by Kern-Godal et al. [31] is consistent with observations of EAS for individuals with PTSD, another population with historically poor treatment outcomes and high attrition rates [33]. The finding that EFP reduces negative psychosocial symptoms, specifically anxious symptomatology, as reported by Atherton et al. [25], has also been reported in non-SUD populations [14–16]. Considering that mental illness may lead to the persistence of SUDs through mechanisms such as self-medication of dysphoric mood [34], it is potentially worth investigating whether EAS could be useful adjuncts in the treatment of those dually diagnosed with mental health conditions and SUDs.

Qualitative studies highlight a number of beneficial effects of EAS for individuals in treatment for and/or diagnosed with SUDs. Qualitative findings are consistent with those of other studies investigating EAS as adjunctive therapies for populations with mental health disorders or trauma, where EAS participants have similarly reported decreases in negative psychosocial symptoms and forming bonds with their horses [11, 14, 16, 35]. Yet, there are several limitations to consider regarding the applicability of these results. First, included studies appear to have limited their focus to positive attributes of EAS, indicating potential bias. Three studies specifically made note of this positive/negative imbalance, stating that a pro-horse background among authors may have influenced interpretation of participants’ responses [27–29]. It is equally important to recognize that the themes identified in these studies were limited to adolescent and young adult populations.

### Directions for future research

#### Quantitative studies

Future research into EAS programs should focus on methodologically rigorous studies. While the field would benefit from additional RCTs with sufficient statistical power, there is also a role for non-randomized prospective cohort studies that allow for easier implementation and further exploration of outcomes impacted by EAS participation, including measures of substance use (e.g., self-report and/or urine drug screens). RCTs moving forward should include careful consideration of TAU conditions and consider potentially incorporating elements (e.g., gardening activities) that control for peripheral aspects of EAS, such as positive effects stemming from being outdoors or engaging in productive work. Furthermore, consideration should be given to three-armed randomized controlled studies that include alternative animal-assisted therapies. Studies of this nature would allow investigators to better isolate EAS-specific effects and the unique contributions of the horse. Additionally, as acknowledged by Gatti et al. [30] who had 7 participants withdraw consent based on randomization assignment, it is reasonable to assume that EAS would be most effective for those actively seeking this type of treatment. Therefore, innovative randomized experimental designs that take into consideration patient preference may more accurately reflect real world implementation and effectiveness [36]. Additionally, however, these observed dropouts may have also been the result of randomizing individuals in a single treatment setting where participants are acutely aware of counterparts’ differing treatment conditions. As such, if multiple residential settings are available, it may be more appropriate to employ cluster randomized trials or stepped-wedge designs where all participants in a single setting receive the same intervention. Independent of exact study design, however, quantitative studies investigating the impact of EAS in populations with SUDs should incorporate larger sample sizes to account for the high probability of dropout in these populations [2] and to enhance the statistical power of findings.

#### Qualitative studies

In addition to quantitative studies, there is an equal need for continued qualitative studies. Qualitative studies are important for further identifying predictors and outcomes that should be rigorously assessed in future quantitative studies. Several themes have already emerged across existing qualitative studies that should inform future study measurements, including improved self-esteem/efficacy and emotional effect/mental health. Moving forward, however, qualitative studies should include more direct exploration of negative participant experiences. As previously described, qualitative studies to this point have seemingly limited their focus to positive attributes of EAS. Evaluating negative perspectives on EAS through intentional assessment (e.g., targeted interview questions) is necessary in order to accurately evaluate the acceptability of EAS among populations in SUD treatment. Additionally, future qualitive studies should consider assessing individuals’ reasons for declining EAS participation (if they consent to this sub-study). Ultimately, qualitative studies will be essential for elucidating possible mechanism of change for EAS as adjuncts to SUD treatment.

#### Standardized terminology for equine interventions

When referencing human services incorporating equines, in both popular media and academic literature, there exist widespread usage of unclear and imprecise terms [6]. This lack of standardization impedes replicable research and creates an environment where EAS interventions cannot be properly evaluated and scientifically developed [37]. Future researchers should work to standardize and optimize terminology surrounding human services incorporating equines. Woods et al*.* [6] provided a starting point to this end, but acknowledge that their recommendations were developed from consensus rather than unanimous decision making and identify their article as a living document. Prior to the resolution of terminology concerns, researchers should provide detailed protocol descriptions such that replicability is possible independent of naming convention [37].

#### Reporting of participants’ demographics

Future research into the effectiveness of EAS in SUD treatment should include improved reporting on study participants’ demographics. Of the nine studies included in this review, less than half provided racial or ethnic demographic information for their study populations [24–26, 29]. Without clear reporting on such characteristics, the generalizability of results across racial and ethnic groups cannot be determined. This is especially significant given the well-documented racial disparities that exist in SUD treatment completion [38–40]. Moreover, four studies provided no breakdown of participants’ substance use [23, 25, 27, 28]. If the impact of EAS on SUD treatment is to be understood, detailed characterization of study participants’ substance use, including identification of SUD diagnosis status and specific SUD, must be reported, and where possible, sub-analyzed. Additionally, we recommend that future research carefully describe treatment settings and concomitant interventions so that results can be appropriately interpreted. Understanding the variance in effect depending on type of SUD or treatment setting will be important in identifying groups that potentially stand to benefit the most from participation in EAS as a part of SUD recovery.

### Limitations

The current scoping review has several limitations. First, the majority of studies included in this review had small sample sizes and likely lacked the statistical power necessary to accurately describe the effect of EAS participation on treatment-related outcomes. Second, summarized results may be subject to publication bias as researchers may have missed unpublished findings of existing EAS programs for individuals with SUD. Finally, the studies included in this review, which provided study location, came from Norway (*n* = 5), Canada (*n* = 2), or the United States (*n* = 1), limiting the generalizability of the findings to those regions. Further, as all nine studies involved exclusively adolescent or young-adult populations, both qualitative and quantitative findings cannot be generalized to older adult populations with SUD.

## Conclusions

Despite suggestive positive trends, methodological limitations, limited population diversity, and an overall dearth of available studies delay conclusions on the effects of EAS as adjuncts to SUD treatment. In addition to a greater number of quantitative and qualitative studies of rigorous design that further explore outcomes impacted by EAS, future research should ensure clear reporting of participant demographics as well as attempt to standardize and optimize terminology related to services incorporating equines. Additionally, future studies should investigate EAS as adjuncts to SUD treatment among older adults and populations in additional geographic locations.

## Supplementary Information

**Additional file 1:** **Supplemental Table 1.** Equine-assisted services for individuals with substance use disorders: a scoping review– Search strategy. **Supplemental Table 2. **Demographic characteristics of studies assessing equine-assisted services in substance use disorder treatment.

## Data Availability

The datasets used and/or analyzed during the current study are available from the corresponding author on reasonable request.
