# Supplementary material for: Equine-assisted services for individuals with substance use disorders: a scoping review
Source: Subst Abuse Treat Prev Policy. 2022 Dec 14;17:81. doi: 10.1186/s13011-022-00506-x (PMC9749232; doi:10.1186/s13011-022-00506-x)
Supplement: Supplementary file 1 — Additional file 1: Supplemental Table 1. Equine-assisted services for individuals with substance use disorders: a scoping review– Search strategy. Supplemental Table 2. Demographic characteristics of studies assessing equine-assisted services in substance use disorder treatment. [file 13011_2022_506_MOESM1_ESM.docx]

**Supplemental Table 1. Equine-assisted services for individuals with substance use disorders: a scoping review– Search strategy**

| **MEDLINE (PubMed) (1966-7/27/2021) (n=7)** | | | |
| --- | --- | --- | --- |
| **Search ID#** | **Query Terms** | | **Results** |
| 1 | Substance-related disorders [MeSH Major Topic] | | 221,433 |
| 2 | ((("Substance use disorder treatment") OR ("Substance abuse treatment")) OR ("Substance dependence treatment")) OR ("Addiction treatment") | | 4,164 |
| 3 | 1 OR 2 | | 222,852 |
| 4 | ((((((("Equine related treatment") OR ("Equine assisted learning")) OR ("Equine assisted therapy")) OR ("Equine assisted psychotherapy")) OR ("Horse assisted therapy")) OR ("Equine facilitated psychotherapy")) OR ("Equine facilitated learning")) OR ("Equine Therapy") | | 2,953 |
| 5 | 3 AND 4 | | 7 |
| **APA PsychINFO (WebLUIS) (1927-7/27/2021) (n=4)** | | | |
| **Search ID#** | | **Query Terms** | **Results** |
| 1 | | MA Substance-related disorders | 37,140 |
| 2 | | "Substance use disorder treatment" OR "Substance abuse treatment" OR "Substance dependence treatment" OR "Addiction treatment" | 15,955 |
| 3 | | S1 OR S2 | 48,029 |
| 4 | | "Equine related treatment" OR "Equine assisted learning" OR "Equine assisted therapy" OR "Equine assisted psychotherapy" OR "Horse assisted therapy" OR "Equine facilitated psychotherapy" OR "Equine facilitated learning" OR "Equine Therapy" | 272 |
| 5 | | S3 AND S4 | 4 |
| **CINAHL Plus with Full Text (1961-7/27/2021) (n=2)** | | | |
| **Search ID#** | | **Query Terms** | **Results** |
| 1 | | substance-related disorders OR substance related disorders | 31,054 |
| 2 | | "Substance use disorder treatment" OR "substance abuse treatment" OR "Substance dependence treatment" OR "Addiction treatment" | 6,104 |
| 3 | | 1 OR 2 | 35.018 |
| 4 | | "Equine related treatment" OR Equine assisted learning" OR "Equine assisted therapy" OR "Equine assisted psychotherapy" OR "Horse assisted therapy " OR "Equine facilitated psychotherapy" OR "Equine facilitated learning" OR "Equine Therapy" | 671 |
| 5 | | 3 AND 4 | 2 |
| **Academic OneFile (1995-7/27/2021) (n=179)** | | | |
| Search ID# | | Query Terms | Results |
| 1 | | Advanced Search: Keyword: "Equine related treatment" OR Keyword: "Equine assisted learning" OR Keyword: "Equine assisted therapy" OR Keyword: "Horse assisted therapy" OR Keyword: "Equine facilitated psychotherapy" OR Keyword: "Equine facilitated learning" OR Keyword: "Equine therapy" AND Keyword: "Substance use disorders" | 179 |

NOTE: The search engine for Academic OneFile only allows for 10 search rows and does not allow the user to combine searches in the same way as the other included search engines. Authors conducted a search of all equine-related keywords and added “substance use disorders” to focus entries.

**Supplemental Table 2. Demographic characteristics of studies assessing equine-assisted services in substance use disorder treatment**

| Author, Year | Age | Sex | Race/Ethnicity | Substance Use | Comorbid Conditions |
| --- | --- | --- | --- | --- | --- |
| Adams et al.^1^ | Mean Age: 15 | Female – 100% | First Nations- 100% | Volatile Substances- 100% | *Not Provided* |
| Atherton et al.^2^ | Age Range: 14-17 | Male- 60%  Female- 40% | African American- 50%  Caucasian- 40%  Biracial- 10% | Not Provided | *Not Provided* |
| Brenna^3^ | Mean Age: 24.75 | Male- 50%  Female- 50% | *Not Provided* | *Not Provided* | *Not Provided* |
| Dell et al.^4^ | Mean Age:  Boys- 14  Girls- 15 | Male – 47%  Female - 53% | Boys:  First Nation- 71%  Inuit- 29%  Girls:  First Nation- 75%  Other- 25% | Boys  Solvents- 100%  Alcohol and Other Substances- 86%  Girls:  Solvents- 100%  Other Substances- 88% | *Not Provided* |
| Gatti et al.^5a,b^ | Year < 20- 8%  20 ≤ yea ≤26- 78%  26 ≤ yea ≤ 30- 14% | Male – 60%  Female – 40% | *Not Provided* | Cannabis  Alcohol  Heroin  Amphetamine  Benzodiazepine  GHB  Cocaine | Mood Disorders  Neurotic Stress/Post-Traumatic Stress Disorders  Personality Disorders  Behavioral and Emotional Disorders |
| Kern-Godal et al.^6c^ | Mean Age – 23.1 | Male – 72.2%  Female – 27.8% | *Not Provided* | Cannabis- 38.9%  Alcohol- 18.5%  Heroin- 15.7%  Amphetamine- 13.9%  Benzodiazepine- 7.4%  GHB- 3.7%  Cocaine- 1.9% | Behavioral Disorders- 36.2%  Neurotic/Stress Disorders- 34.8%  Mood Disorders- 20.3%  Other Disorders- 8.7% |
| Kern-Godal, Brenna, Arnevik, et al.^7^ | Mean Age- 24.8 | Male- 50%  Female- 50% | *Not Provided* | *Not Provided* | *Not Provided* |
| Kern-Godal, Brenna, Kogstad, et al.^8^ | Mean Age- 25 | Male- 50%  Female- 50% | *Not Provided* | *Not Provided* | ADHD- 25%  Depression- 12.5%  Personality Disorder- 12.5% |
| Stiltner^9^ | Mean Age- 15.6 | Male – 100% | American Indian- 25%  European American- 62.5%  Hispanic American- 12.5% | Marijuana- 87.5%  Alcohol- 12.5%  Poly substances- 12.5%  Opiates- 12.5%  Benzodiazepines- 12.5%  Xanax- 12.5%  Methamphetamine- 25%  K2- 12.5%  Pills- 12.5% | Oppositional Defiant Disorder- 37.5%  ADHD- 50%  Depression- 37.5%  Anxiety- 37.5% |

^a^ Percentage of substances used among participants not provided

^b^ Percentage of comorbid conditions among participants not provided

^c^ Percentages of substances used among study participants refer to primary substance of use

**References**

1. Adams C, Arratoon C, Boucher J, Cartier G, Chalmers D, Dell CA, et al. The Helping Horse: How Equine Assisted Learning Contributes to the Wellbeing of First Nations Youth in Treatment for Volatile Substance Misuse. Hum Anim Interact Bull. 2015;1(1):52–75.
2. Atherton WL, Meola CC, Pritchard KS. Innovative Equine Facilitated Psychotherapy Intervention for Adolescent Addiction Treatment: A Pilot Study. Int J High Risk Behav Addict. 2020;9(3). doi:10.5812/ijhrba.103877
3. Brenna IH. “They are a part of what made my treatment positive. And maybe more meaningful.”: Participants’ experiences of horse-assisted therapy in addiction treatment. [master’s thesis on the internet]. Oslo (NO): University of Oslo; 2013 [cited 2021 Jul 27]. Available from: <https://www.duo.uio.no/bitstream/handle/10852/36778/MA_Ida_Halvorsen_Brenna%5B1%5D.pdf?isAllowed=y&sequence=1>
4. Dell CA, Chalmers D, Bresette N, Swain S, Rankin D, Hopkins C. A Healing Space: The Experiences of First Nations and Inuit Youth with Equine-Assisted Learning (EAL). Child Youth Care Forum. 2011;40(4):319–36. doi: 10.1007/s10566-011-9140-z
5. Gatti F, Walderhaug E, Kern-Godal A, Lysell J, Arnevik EA. Complementary horse-assisted therapy for substance use disorders: a randomized controlled trial. Addict Sci Clin Pract. 2020;15(1):7. doi: 10.1186/s13722-020-0183-z
6. Kern-Godal A, Arnevik EA, Walderhaug E, Ravndal E. Substance use disorder treatment retention and completion: a prospective study of horse-assisted therapy (HAT) for young adults. Addict Sci Clin Pract. 2015;10:21. doi: 10.1186/s13722-015-0043-4
7. Kern-Godal A, Brenna IH, Arnevik EA, Ravndal E. More Than Just a Break from Treatment: How Substance Use Disorder Patients Experience the Stable Environment in Horse-Assisted Therapy. Subst Abuse. 2016;10:99–108. doi: 10.4137/SART.S40475
8. Kern-Godal A, Brenna IH, Kogstad N, Arnevik EA, Ravndal E. Contribution of the patient-horse relationship to substance use disorder treatment: Patients’ experiences. Int J Qual Stud Health Well-being. 2016;11:31636. doi: 10.3402/qhw.v11.31636
9. Stiltner C. Equine-assisted psychotherapy in residential substance use treatment program for male adolescents [dissertation on the internet]. Ann Arbor (MI): University of Capella; 2013 [cited 2021 Jul 27]. Available from: <https://www.proquest.com/openview/761e6c34dd551a4b1949d9151c81f038/1.pdf?pq-origsite=gscholar&cbl=18750>
